# Excess A-subunits of Shiga toxin 2a are produced in enterohemorrhagic *Escherichia coli*

**DOI:** 10.1038/s41598-025-01342-2

**Published:** 2025-05-14

**Authors:** Katrin Neudek, Theresa Kunz, Holger Barth, Herbert Schmidt

**Affiliations:** 1 Department of Food Microbiology and Hygiene, Institute of Food Science and Biotechnology, University of Hohenheim, Garbenstrasse 28, 70599 Stuttgart, Germany; 2 Institute of Experimental and Clinical Pharmacology, Toxicology and Pharmacology of Natural Products, University of Ulm Medical Center, Albert-Einstein-Allee 11, 89081 Ulm, Germany

**Keywords:** Shiga toxins, Enterohemorrhagic *Escherichia coli*, AB_5_ toxins, Gene expression, Toxin production, Pathogens, Bacterial genetics

## Abstract

Shiga toxins (Stx) produced by Shiga toxin-producing *Escherichia coli* (STEC) and enterohemorrhagic *E. coli* (EHEC) are ribosome-inactivating AB_5_ proteins that consist of one enzymatic active A-subunit (StxA) and a pentamer of non-covalently linked B-subunits (StxB). The description of Stx as an AB_5_ protein and the observation that A-subunits without their corresponding B-subunits also intoxicate eukaryotic cells, led to the question whether A- and B-subunits are produced in the bacteria in a 1:5 ratio or whether the A-subunit of the clinically most prominent subtype Stx2a is transcribed in excess revealing free A-subunits released in the bacterial environment. The aim of this study was therefore, to investigate the genetic and protein-based background for this observation in six Stx2a-encoding STEC and EHEC wildtype strains. For this purpose, transcriptional analysis of the Stx2a subunit genes, *stxA2a* and *stxB2a*, was performed by quantitative real-time PCR in one foodborne O113:H21 STEC isolate (strain TS18/08) and five HUS-associated EHEC strains with the serotypes O157:H7/H^−^ (HUSEC003, HUSEC004), O103:H^−^ (HUSEC008), O26:H11 (HUSEC018), and O104:H4 (LB226692). Contrary to the hypothesis that the A- and B-subunit genes are expressed in a ratio of 1:5 comparable to the holotoxin structure or in a ratio of 1:1 based on the operon structure, the results showed that *stxA2a* was expressed 1.90 ± 0.55-times stronger than the gene encoding the B-subunit, possibly indicating the presence of free A-subunits. In addition, strain-specific differences regarding the mRNA fold-changes of the A-subunit gene were observed. By use of native polyacrylamide gel electrophoresis and subsequent Western blot analysis, those single A-subunits were indeed detected in the culture supernatants of all six strains. To investigate whether the transcription ratios between A- and B-subunits observed are in a similar range as the amount of subunit proteins present after translation, a quantitative ELISA specific for StxA2a and StxB2a was established. Quantification of the subunits on protein level by use of ELISA revealed that the subunit ratio of StxA2a:StxB2a is 1.10 ± 0.20 for the strains HUSEC003, HUSEC004 and HUSEC008, but 4.63 ± 0.31 for the strains TS18/08, LB226692, and HUSEC018. The results of this study demonstrated that on both, the transcriptional and the translational level, the established 1:5 subunit ratio is not present in all investigated strains. In addition, the ratios observed after translation indicate that in some strains StxA2a subunits are even produced in higher amounts than B-subunits.

**Supplementary Information:**

The online version contains supplementary material available at 10.1038/s41598-025-01342-2.

## Introduction

Shiga toxin-producing *Escherichia coli* (STEC) can be categorized as a group of pathogenic *E. coli* capable to produce one or more Shiga toxins (Stx), and which may cause human disease^1^. Most isolates were found in human and animal feces, food and water^2–7^. The term enterohemorrhagic *E. coli* (EHEC) has been introduced for those STEC that cause serious symptoms in humans such as abdominal pain, watery to bloody diarrhea and hemorrhagic colitis^8–11^. In 5–10% of infections, patients develop the hemolytic-uremic syndrome which is characterized by hemolysis, thrombocytopenia, and glomerular kidney lesion^9,12–15^.

The major pathogenicity factor of EHEC is the production of one or more Stx. Generally, Stx can be classified into two groups: Stx1 and Stx2. The toxins within both groups vary in their amino acid sequence and toxicity to different cell lines and animal models^16–21^. Within these groups, Stx can be further divided into different subtypes. The more homogenous Stx1 group comprises the subtypes Stx1a, Stx1c, Stx1d and Stx1e (the latter has been described in *Enterobacter cloacae*)^22–27^. All those subtypes are similar to the toxin produced by *Shigella dysenteriae* type 1. The Stx2 group is a more heterogenous group and comprises the subtypes Stx2a-Stx2m^28–38^. While Stx1 only rarely leads to severe symptoms, members of the Stx2 group, especially Stx2a and Stx2c, are more often associated with severe diseases^12,39^.

Stx are AB_5_-type protein toxins consisting of one enzymatically active A-subunit and a pentamer of non-covalently linked B-subunits^40–43^. The A-subunit consists of two domains: the catalytic A_1_ and the A_2_ domain, the latter consisting of an α-helix responsible for holotoxin assembly. Both domains are linked via a disulfide bond^44^. The catalytic domain exhibits a rRNA-*N*-glycosidase activity, resulting in the depurination of the 28S ribosomal subunit at the position 4324 in eukaryotic cells^45,46^. As a consequence, the binding of amino-acyl-tRNAs to the ribosome is inhibited leading to ribocytotoxic stress and apoptosis of the target cell^45,47^. The pentameric B-subunit is responsible for binding to the receptor globotriaosylceramide (Gb3), which is located on the plasma membrane of target cells such as microvascular endothelial cells present in kidneys, brain and other organs^48–50^.

**Fig. 1:**
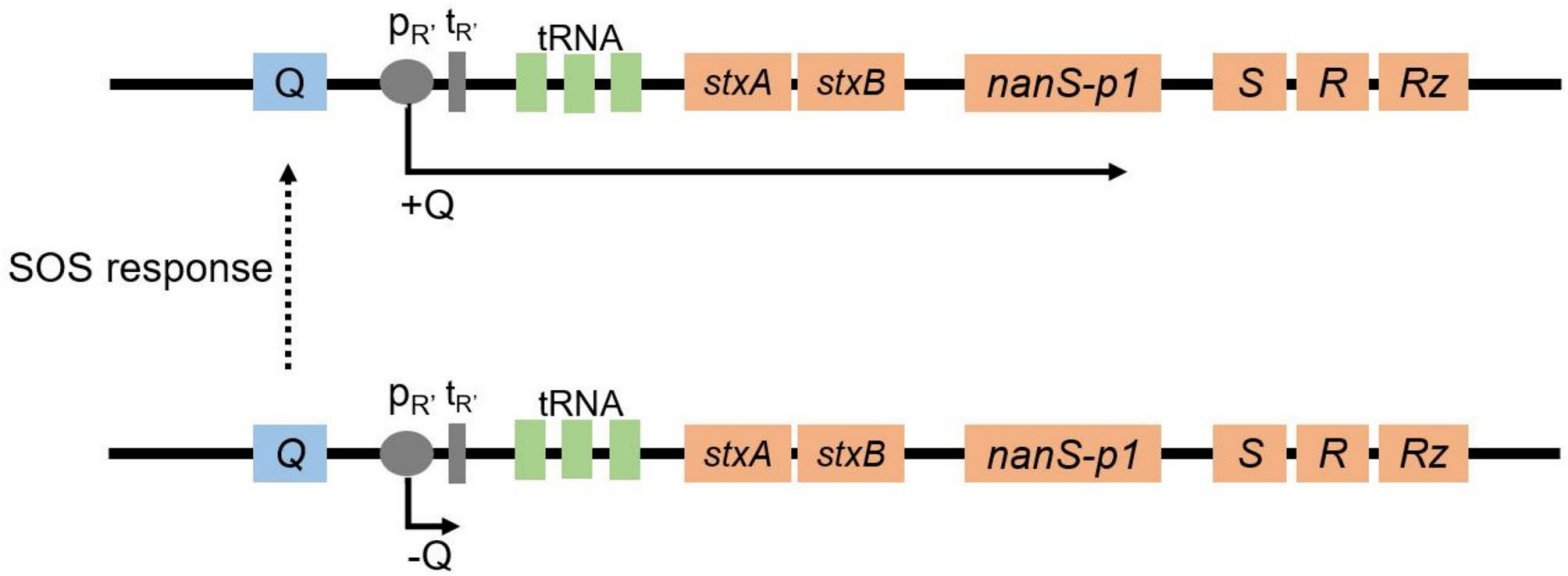
Scheme of the genomic structure of the late-regulated region in the *stx2a*-phage 933W. Region consists of the antiterminator gene *Q* (blue), and the *stx*_2a_ operon with the subunit genes *stxA2a* and *stxB2a*, the *nanS-p1* gene and the genes encoding the phage lysis cassette *S*, *R*, and *Rz* (orange). Transcription starts at the promoter pR’ (gray). Upon induction of bacterial SOS response, expression of the antiterminator Q allows the RNA polymerase to read through the terminator tR’ (grey) and thereby transcription of the *stx2a* operon^51,52^.

The Stx2a subunits are encoded by the genes *stxA2a* and *stxB2a* in an operon structure within the genome of lambdoid prophages. In the most prominent *stx2a* phage 933W both genes are located downstream of the gene encoding the antiterminator Q, the promoter pR’, the terminator tR, and the genes encoding the methionyl-tRNA and the arginyl-tRNA^51,52^. Downstream of the subunit genes, the *nanS-p1* gene and the genes encoding the phage lysis cassette, namely *S*, *R* and *Rz* are located^51^. The transcription of the genes in the late-regulated phage region depends on the expression of the antiterminator gene Q (Fig. 1)^53–55^. When the bacteria grow under normal conditions, the antiterminator Q is not expressed and transcription of the late-regulated phage region takes place from the promoter pR’ to the terminator tR, resulting in a short transcript with no genetic information (Fig. 1)^56^. Upon induction (e.g., UV-radiation), the antiterminator Q is expressed (Fig. 1), and allows the RNA polymerase to read through the terminator, resulting in the expression of the Stx subunit genes and the lysis of the bacterial host cell^53^. This appears to be a general regulatory structure present in many Stx-phages^57,58^.

Stx has been assumed in the literature to act as holotoxin in its AB_5_ assembled structure^59–62^. However, Heinisch et al.^63^ showed that the subunit StxA2a is cytotoxic to HeLa, Vero B4, and HCT-116 cells even in absence of its corresponding B-subunits. The same was shown by Funk et al.^64^ for the subtilase cytotoxin, which is also an *E. coli* AB_5_ protein toxin. In addition, Sessler et al.^65^ observed that the A-subunit of AB_5_ protein toxins can be taken up into target cells and retrogradely transported to the endoplasmic reticulum in the absence of B-subunits. Since all those studies were performed with purified toxin subunits, the aim of the present study was to clarify whether there is a genetic and protein-based background for these observations in STEC and EHEC wildtype strains. Based on the AB_5_ structure of the holotoxin it was assumed that both subunit genes are transcribed and translated in a 1:5 ratio. In contrast, the operon structure of the Stx region might lead to a polycistronic transcript, resulting in a 1:1 ratio. Such a polycistronic transcript was also suggested by Sung et al.^66^. To investigate this hypothesis, EHEC strains with a strong clinical background were used. Most of these strains derived from the HUSEC strain collection containing representative strains from HUS patients^67^. Moreover, the highly pathogenic O104:H4 outbreak strain from 2011^68^ and a food isolate were used^7^.

## Results

### Unexpectedly, the Stx2a subunit genes *stxA2a* and *stxB2a* are not transcribed in a 1:5 or 1:1 ratio

**Fig. 2:**
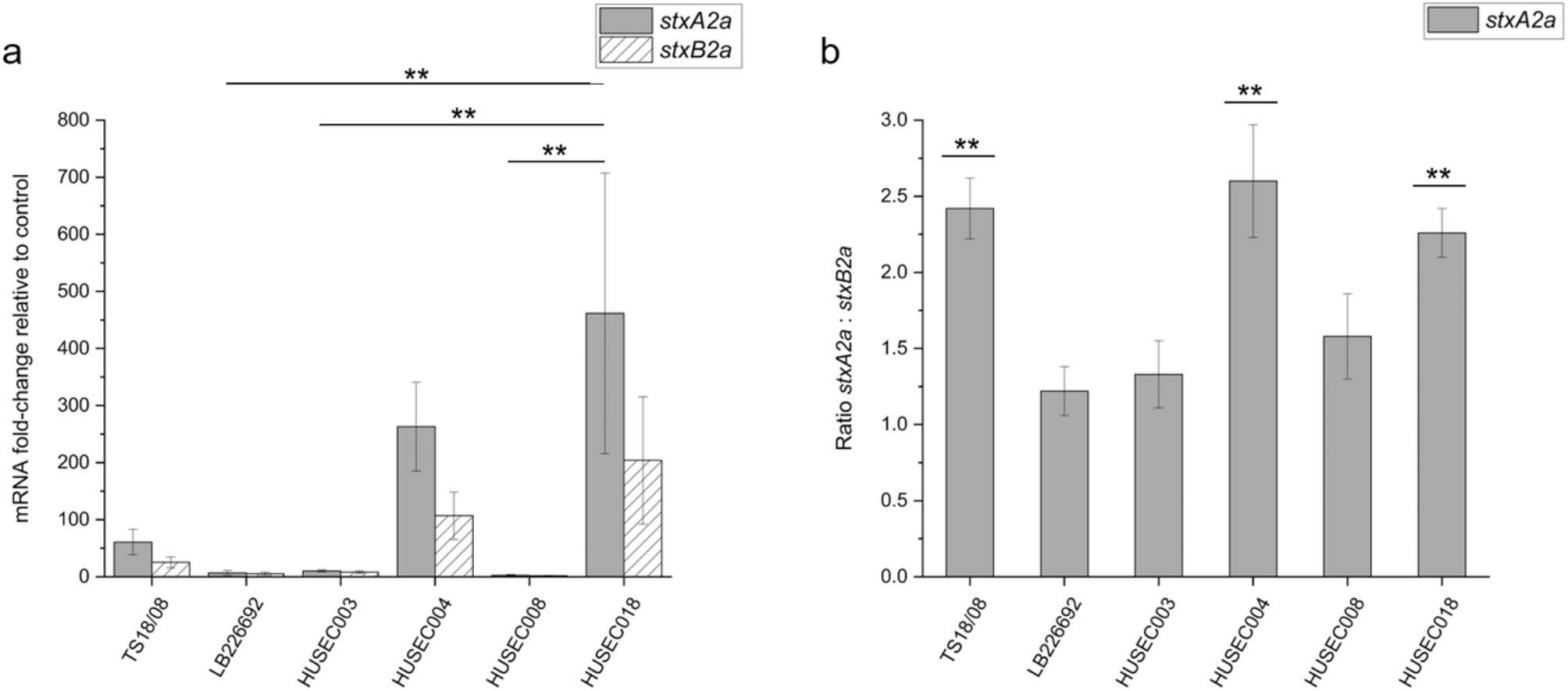
Relative normalized expression (**a**) and expression ratio (**b**) of *stxA2a* and *stxB2a* in *E. coli* strains TS18/08, LB226692, HUSEC003, HUSEC004, HUSEC008, and HUSEC018. Relative normalized transcription is shown as fold-change relative to the level of the respective non-induced cultures. Expression ratio was calculated from mRNA fold-change of *stxA2a* and *stxB2a*. Gene expression was investigated under inducing (100 ng/mL norfloxacin) and non-inducing growth conditions using qPCR. Quantification was performed using *rrsB* as an endogenous control. Data are means ± standard deviation of the experiments performed in three biological replicates (n = 3). Statical analysis was performed with the following tests: Shapiro–Wilk test, Tukey test and Bonferroni test. Significance in (**b**) indicates comparison to *stxB*2a:*stxB*2a ratio. Asterisks (**) indicate significance in subunit gene expression (*p* < 0.01).

To investigate Stx2a subunit gene expression, transcriptional analysis was performed for six Stx2a-encoding STEC and EHEC wildtype strains by quantitative real-time PCR (qPCR). The gene expression of the subunit genes *stxA2a* and *stxB2a* was analyzed and normalized to the expression of the house-keeping reference gene *rrsB*. For each strain, gene expression of the norfloxacin-induced culture was compared to the corresponding gene expression values of the non-induced culture. In order to assess the subunit ratio on mRNA level more precisely, the ratio between mRNA-fold change of *stxA2a* and *stxB2a* was calculated for each strain respectively (Fig. 2, Table S1).

Overall, the mRNA fold-change values showed differences between the investigated strains (Fig. 2a, Table S1). *Escherichia coli* O26:H11 strain HUSEC018 and *E. coli* O157:H^−^ strain HUSEC004 showed highest expression of both subunit genes (~ 450-fold and ~ 250-fold higher expression of *stxA2a*; ~ 200-fold and ~ 100-fold higher expression of *stxB2a*, respectively) when compared to the non-induced culture (Fig. 2a). In contrast, strains HUSEC003, HUSEC008 and the 2011 outbreak strain LB226692 showed only slightly higher expression of Stx subunit genes upon induction (Fig. 2a). For the STEC strain TS18/08, an approximately 60-fold higher expression of *stxA2a* and a ~ 25-fold higher expression of *stxB2a* was observed (Fig. 2a). Regarding the subunit gene ratios, the strains HUSEC004, TS18/08, and HUSEC018 showed the highest with values > 2.00 (2.60 ± 0.37, 2.42 ± 0.20, and 2.26 ± 0.16, respectively), meaning that the gene encoding StxA2a was significantly higher expressed (n = 3, *p* < 0.01) than the gene encoding StxB2a (Fig. 2b). The other strains showed values between 1.22 ± 0.16 for strain LB226692 and 1.58 ± 0.26 for strain HUSEC008 (Fig. 2b). Here, a higher *stxA2a* expression but no significant difference was observed. The results obtained by qPCR revealed that the overall expression levels of the Stx subunit genes differed among the investigated Stx2a-encoding STEC and EHEC strains. Calculation of the transcription ratio of *stxA2a*:*stxB2a* showed a higher expression of *stxA2a* in all six Stx2a-encoding strains with a mean transcription ratio of 1.90 ± 0.55.

### Free Stx subunits are detectable in culture supernatants of STEC and EHEC wildtype strains

The results of the transcriptional analysis demonstrated a higher expression of *stxA2a* in all investigated strains, raising the question whether the subunit protein levels of StxA2a are also higher. For this purpose, culture supernatants of all strains were collected 24 h after induction with norfloxacin and concentrated. Subsequently, native PAGE and subsequent Western blot analysis were performed.

**Fig. 3:**
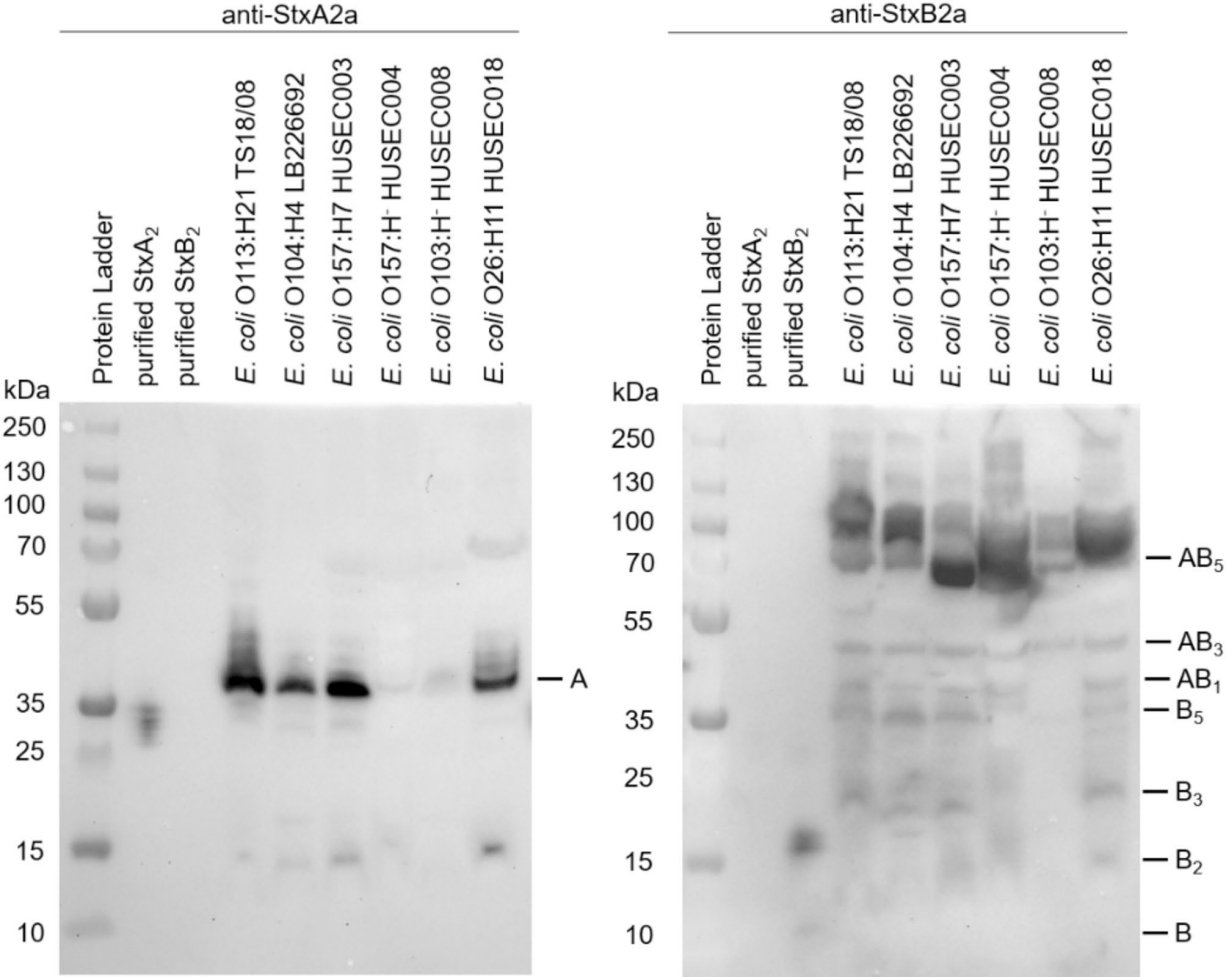
Western blot analysis of culture supernatants of *E. coli* strains TS18/08, LB226692, HUSEC003, HUSEC004, HUSEC008, and HUSEC018. Samples were collected using centrifugal filter units (MWCO: 3000 Da). PageRuler Plus Prestained Protein ladder (Thermo Fisher Scientific), purified StxA_2_ and StxB_2_ and concentrated culture supernatants were applied to Bolt 4–12% Bis–Tris Plus gels. Western blot was detected with subunit-specific antibodies (Shiga toxin 2A monoclonal antibody (11E10) and anti-SCH-SKY antibody).

The Shiga toxin 2A monoclonal antibody (11E10) detected the single A-subunits (~ 35 kDa) in all six Stx2a-encoding STEC and EHEC strains, with higher protein levels for *E. coli* strains TS18/08, LB226692, HUSEC003 and HUSEC018 (Fig. 3). Detection with the anti-SCH-SKY antibody revealed holotoxin complexes with a size of ~ 70 kDa, but also dimers (~ 15 kDa), trimers (~ 20 kDa), pentamers (~ 35 kDa) and possible other StxA2a-StxB2a subunit complexes (~ 40 kDa and 50 kDa) (Fig. 3). Overall, the Western blot analysis showed that single Stx A-subunits are present in the culture supernatants of the investigated STEC and EHEC wildtype strains and that most B-subunits are bound in holotoxin (AB_5_) complexes. However, as Western blot analysis is not a quantitative method, and the subunit ratio may have been influence by the concentration of the culture supernatants, an ELISA enabling specific quantification of StxA2a and StxB2a was established.

### The subunit ratio (StxA2a:StxB2a) differs between STEC and EHEC wildtype strains

To quantify the protein concentration and the number of A- and B-subunits in the culture supernatants of the six Stx2a-encoding STEC and EHEC wildtype strains, a quantitative ELISA, specific for StxA2a and StxB2a was established (see supplementary material). For this purpose, culture supernatants of all strains were collected at 24 h after induction with norfloxacin and used unconcentrated in the ELISA. For quantification of the subunits in the STEC and EHEC culture supernatants, samples were denatured at 80 °C for 1 h prior to coating to ensure that all subunits are present in their monomeric form. For each strain, the protein concentration of StxA2a and StxB2a was determined and used to calculate the number of subunits (Tables 1 and S2). To assess the stoichiometric ratio, the ratio between StxA2a and StxB2a was calculated for each strain by dividing the number of StxA2a subunits by the number of StxB2a subunits, respectively.

**Table 1:** Protein concentration, number of subunits of StxA2a and StxB2a, and subunit ratios in *E. coli* strains TS18/08, LB226692, HUSEC003, HUSEC004, HUSEC008, and HUSEC018.

Strain	protein concentration [µg/mL]*		number of subunits per mL [× 10^12^]*		subunit ratio [-]
	StxA2a	StxB2a	StxA2a	StxB2a	StxA2a:StxB2a
TS18/08	0.91 ± 0.13	0.05 ± 0.00	17.06 ± 2.38	4.07 ± 0.02	4.19 ± 0.59
LB226692	0.96 ± 0.09	0.05 ± 0.00	17.99 ± 1.76	3.74 ± 0.29	4.19 ± 0.59
HUSEC003	1.29 ± 0.38	0.28 ± 0.01	24.19 ± 7.11	21.88 ± 0.82	1.12 ± 0.37
HUSEC004	0.49 ± 0.06	0.09 ± 0.01	9.27 ± 1.12	7.09 ± 0.72	1.34 ± 0.30
HUSEC008	0.33 ± 0.03	0.09 ± 0.01	6.27 ± 0.51	7.40 ± 0.46	0.85 ± 0.12
HUSEC018	1.58 ± 0.29	0.08 ± 0.02	29.79 ± 5.46	6.59 ± 1.52	4.82 ± 1.47

*Protein concentration, number of subunits, and subunit ratios were investigated under inducing growth conditions (100 ng/mL norfloxacin) using quantitative ELISA. Quantification was performed using a StxA2a and StxB2a standard within the range of 7.8 ng/mL and 1000 ng/mL. Data are means ± standard deviation of the experiments performed in three biological replicates (n = 3).

**Fig. 4:**
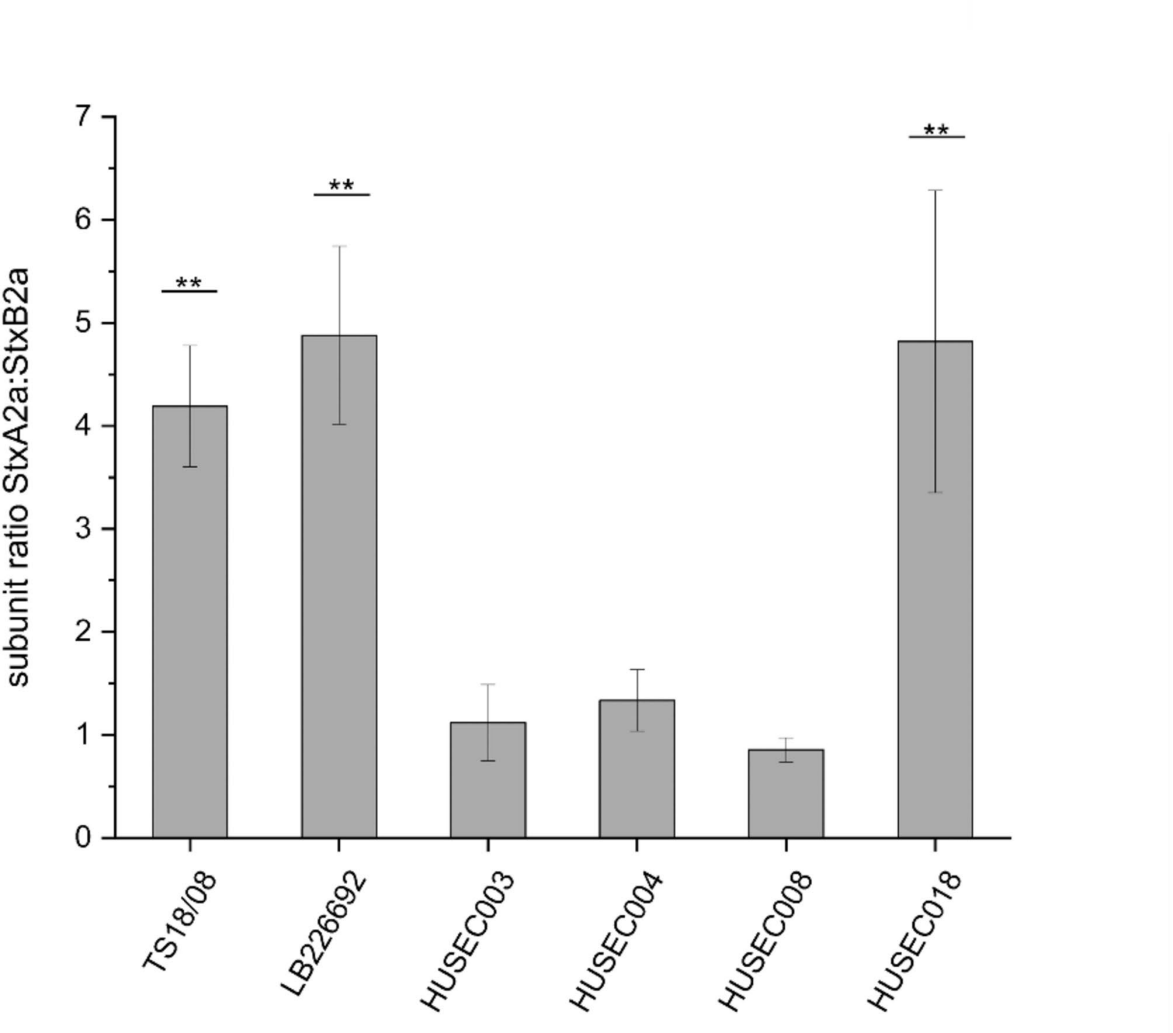
Subunit ratio of StxA2a and StxB2a in *E. coli* strains TS18/08, LB226692, HUSEC003, HUSEC004, HUSEC008, and HUSEC018. Subunit ratio was calculated from number of subunits of StxA2a and StxB2a, investigated under inducing growth conditions (100 ng/mL norfloxacin) using quantitative ELISA. Quantification was performed using a StxA2a and StxB2a standard within the range of 7.8 ng/mL and 1000 ng/mL. Data are means ± standard deviation of the experiments performed in three biological replicates (n = 3). Statical analysis was performed with the following tests: Shapiro–Wilk test, Tukey test and Bonferroni test. Significance in (**b**) indicates comparison to StxB2a:StxB2a ratio. Asterisks (**) indicate significance in subunit gene expression (*p* < 0.01).

Regarding the protein concentration and the number of StxA2a and StxB2a, differences were observed between the six investigated Stx2a-encoding STEC and EHEC wildtype strains. Highest concentration of StxA2a and highest number of subunits was determined for *E. coli* O26:H11 strain HUSEC018 and *E. coli* O157:H7 strain HUSEC003 with 1.58 µg/mL (corresponds to 2.98 × 10^13^ subunits) and 1.29 µg/mL (corresponds to 2.42 × 10^13^ subunits) respectively (Table 1). For STEC isolate O113:H21 strain TS18/08 and *E. coli* O104:H4 strain LB226692 concentrations of approximately 0.9 µg/mL were determined (Table 1). Lowest concentration and number of subunits of StxA2a was measured for strains HUSEC004 and HUSEC008 (Table 1). For StxB2a, the highest concentration and number of subunits was observed with 0.28 µg/mL and 0.09 µg/mL for the strains HUSEC003, HUSEC004 and HUSEC008 respectively (Table 1). In the culture supernatants of the other strains, concentrations between 0.05 µg/mL (strain LB226692) and 0.08 µg/mL (strain HUSEC018) were determined (Table 1). Regarding the subunit ratio (StxA2a:StxB2a), the strains HUSEC003, HUSEC004 and HUSEC008 showed equal subunit ratios with a mean of 1.10 ± 0.20 (Fig. 4, Table S2). Surprisingly, strains TS18/08, LB226692 and HUSEC018 showed values between 4.19 ± 0.59 (TS18/08) and 4.88 ± 0.86 (LB226692) demonstrating that significantly more A-subunits (n = 3, *p* < 0.01) are present in the culture supernatants of those strains (Fig. 4, Table S2).

## Discussion

The formation of one or more Stx is considered to be a major determinant of EHEC-associated pathogenesis. These AB_5_ protein toxins inhibit the protein biosynthesis in the eukaryotic target cells, leading to apoptosis of these cells^45,47–50^. While Stx is described to act as an AB_5_ protein, the studies by Heinisch and Krause et al. and Funk et al.^63,64^ have shown that the A-subunit of both, Stx and subtilase cytotoxin (SubAB), another *E. coli* AB_5_ protein toxin, is cytotoxic to different eukaryotic cell lines even in absence of their corresponding B-subunits. Therefore, the aim of the current study was to investigate the genetic and protein-based background for Stx transcription and translation in STEC and EHEC strains and explain the putative occurrence of free A-subunits. For this purpose, five representative Stx2a-encoding EHEC wildtype strains with different serotypes from the HUSEC collection were selected, since Stx2a is most frequently implicated in severe diseases^12^ and associated with high cytotoxicity and low effective and lethal doses in human cells and animal models^18,21^. Strain TS18/08 was included as a foodborne Stx-producing strain without clinical background.

To analyze the expression of *stxA2a* and *stxB2a*, transcriptional analysis was performed with quantitative real-time PCR and the expression ratios of both genes were calculated. The hypothesis was that the genes encoding the Stx subunits are expressed either in a 1:5 ratio, in concordance with the AB_5_ holotoxin structure, or in a 1:1 ration, due to the operon structure and data from other authors suggesting that the late phase phage genes of lambdoid phages are transcribed in a single mRNA^69–73^. Contrary to this initial hypothesis, the results showed that the *stxA2a* mRNA could be detected in 1.22–2.60-fold excess, when compared to the *stxB2a* mRNA in the investigated strains (Fig. 2). Similar results were obtained in the study by Herold et al.^74^. In this study, the authors showed that the StxA2a subunit gene is more efficiently transcribed than the StxB2a subunit gene in *E. coli* O157:H7 strain EDL933 and observed transcription ratios of 3.88 and 2.09 (*stxA2a*:*stxB2a*) using microarray analysis and qPCR, respectively. Using the same strain, Kijewski et al.^75^ also showed in a recent study by use of transcriptome analysis that transcription of *stxA2a* is ~ 3.5-times higher compared to *stxB2a*. Contrarily to those results, Sy et al.^76^ observed in their RNA-seq study a slightly higher transcription of *stxB2a* compared to *stxA2a* for strain *E. coli* O157:H7 Sakai. Nevertheless, the results of this study together with those of the other studies raise the question whether the genes for both subunits are indeed located on a single mRNA transcript, as polycistronic mRNA, or whether there is a separate transcript for each subunit. The latter might be a possible explanation for the unequal ratio after transcription. However, Sung et al.^66^ demonstrated in their study that both Stx2 subunit genes are transcribed as a polycistronic transcript with a size of approximately 1.6 kb.

The subunit ratios observed in the current study after Stx transcription might indicate that free A-subunits occur in the bacterial environment of STEC and EHEC strains. However, the differences in gene expression might be balanced by processes during translation. Therefore, the presence of free Stx subunits and the ratio of StxA2a:StxB2a in the culture supernatants of the six wildtype strains were examined by Western blot analysis and quantitative ELISA. Results of the Western blot analysis revealed that using the anti-StxA antibody, free A-subunits, with a molecular weight of ~ 35 kDa but no holotoxin complexes were detected in the supernatants of all six strains (Fig. 3). When comparing the bands of the wildtype strains to that of the control, differences in the molecular weights can be observed. Similar differences were shown by Smith et al.^77^. The bands at a higher molecular weight most likely represent the full-length StxA2a with intact signal sequence while the band of the control (purified StxA2a) most likely represents the mature toxin without signal sequence^77^. It is not clear why the Shiga toxin 2A monoclonal antibody used cannot bind to the holotoxin complex. However, the study by Smith et al.^77^ which examined the epitope of the antibody, was also unable to detect any purified Stx2 holotoxin complexes in the Western blot analysis. Regarding the B-subunits, Western blot analysis showed that most B-subunits are bound in holotoxin complexes with a size of ~ 72 kDa or exists in various StxA2a-StxB2a subunit complexes with sizes of ~ 40 kDa and ~ 50 kDa (Fig. 3). According to the study of Donohue-Rolfe et al.^78^ those molecular weights correspond to A-1B, A-2B, A-3B, and A4B complexes. Where these different multimer conformations come from and whether they are products of holotoxin assembly or dissociation could not be determined with the methodology used here. Several studies showed that the Stx2B pentamer assembly is strongly dependent on temperature, subunit concentration, and ionic strength^79–82^. Kitova et al.^82^ demonstrated in their study, that at low concentrations, as observed in this study with approximately 36 µM StxB2a for strain HUSEC003, Stx2B exists preferentially as monomer and dimer. In the same study the authors could not detect free A-subunits originating from the dissociation of the AB_5_ holotoxin^82^. This might mean that the free A-subunits detected with the anti-StxA antibody are not products of the dissociation of the holotoxin complexes. However, since Kitova et al.^82^ used electrospray ionization mass spectrometry and in vitro conditions, the results of their study cannot be directly transferred to interpret those of this study.

To investigate the number of StxA2a and StxB2a subunits in more detail, an ELISA enabling specific quantification of both subunits was established. The results of the ELISA showed strain-specific differences in the concentration of StxA2a and StxB2a, and the corresponding number of subunits. For the strains HUSEC004, and HUSEC008 only low concentrations of StxA2a were observed (Table 1). In contrast, considerably higher concentrations ranging from 0.91 to 1.58 µg/mL were detected in the culture supernatants of strains TS18/08, LB226692, HUSEC003, HUSEC004, and HUSEC018 (Table 1). Regarding StxB2a, highest concentrations were observed for strain HUSEC003 and HUSEC008. In the culture supernatants of the other strains, concentrations below 0.09 µg/mL were determined (Table 1). Thibaut de Sablet et al.^83^ also investigated Stx concentrations in the culture supernatants of Stx2a-encoding strains using ELISA. In their study, concentrations of 5–100 µg/mL were determined in LB broth, 3 h after induction with mitomycin C^83^. However, since induction with mitomycin C causes a significantly greater change in Stx2a production than ciprofloxacin^84^, a fluoroquinolone similar to norfloxacin, the values cannot be directly compared. Interestingly, the concentration and number of Stx subunits determined after translation do not correlate with the mRNA fold-changes observed after transcription. Strains that showed low fold-changes and subunit ratios on mRNA level showed high concentrations on protein level (strains HUSEC003 and LB226692). On the other hand, a low protein concentration was determined for EHEC strain HUSEC004, which showed to have a high mRNA fold-change. Only for the strainsTS18/08 and HUSEC018, both, the mRNA fold-change and the protein concentration correlate with each other and were in the high range.

Based on the calculated number of StxA2a and StxB2a, the subunit ratio after translation was calculated for each strain and here also, the strain-specific differences were apparent. While three of the strains showed equal subunit ratios with a mean of 1.10 ± 0.20 (StxA2a:StxB2a), a ratio of 4.63 ± 0.31 was observed for *E. coli* O113:H21 strain TS18/08, *E.* *coli* O104:H4 strain LB226692 and *E. coli* O26:H11 strain HUSEC018 (Fig. 4). Kijewski et al.^75^ also investigated the fold-changes of StxA2a and StxB2a in *E.coli* O157:H7 strain EDL933 by proteomic analysis. In their study, they observed a fold-change of 3.8 and 6.3 for StxA2a and 9.4 and 56.1 for StxB2a, 3 h and 12 after induction with ciprofloxacin, respectively. Those values result in subunit ratios of approximately 1:1.5 and 1:6 (StxA2a:StxB2a)^75^. The reasons for the differences in the subunit ratios on protein level might be due differences in the type of sample and the timepoint of sampling. While only culture supernatants of the STEC and EHEC strains were examined in this study, Kijewski et al.^75^ used both, the cell lysates and the supernatants. In addition, the samples used in this study were taken 24 h after induction with norfloxacin and not 3 h and 12 h after induction with ciprofloxacin. However, the large differences in the fold-change of StxB2a shown by Kijewski et al.^75^ between 3 h and 12 h after induction might mean that considerable differences could also arise after further 12 h of incubation.

For two of the strains used in this study, namely *E. coli* O104:H4 strain LB226692 and *E. coli* O26:H11 strain HUSEC018, the subunit ratios determined in the culture supernatants were not surprising. The strain LB226692 is a hybrid of enteroaggregative *E. coli* and EHEC and the outbreak strain of the EHEC outbreak in Germany in 2011 causing over 830 cases of HUS and 46 deaths^68,85^. However, strains of the serotype O104:H4 are generally not associated with the development of severe disease^68,85^. *Escherichia coli* strains of the serotype O26:H11, on the other hand, are most frequently reported in association with HUS in the EU^12,39^ and are highly cytotoxic compared to strains of other serotypes^21^. For the *E. coli* strain TS18/08, the results of transcriptional analysis and quantitative ELISA were rather unexpected. This strain was isolated from minced meat in the study by Slanec et al.^7^ and has not yet caused any disease in humans. However, the study by Hauser et al.^86^ showed that the cytotoxicity of strain TS18/08 towards Vero B4 cells was comparable to that of *E. coli* O157:H7 strain EDL933. In general, strains of the serotype O113:H21 are increasingly detected in food and other environmental samples and no clear differences between pathogenic and environmental strains can be observed^87–90^.

In conclusion, both, transcriptional analysis and quantitative ELISA showed that the Stx2a subunits are neither expressed nor produced in the expected 1:5 ratio. The reasons for the strain-specific discrepancy between the findings on transcriptional level and those on translational level are not fully understood but may be related in part to the different regulatory mechanisms on mRNA and protein level and to the unique Stx loci and genomic profiles of the investigated strains. The ratios observed after translation, strongly indicate that free A-subunits are present in the culture supernatants of the six investigated Stx2a-encoding STEC and EHEC strains, which do not find B-subunits for holotoxin assembly. Several mechanisms that might be responsible for the overproduction of StxA2a can be discussed. At the transcriptional level, promoters that exist in the *stx*-operon in addition to pR’ might influence the transcription levels of the single subunit genes. In the *slt-I*-operon (syn. *stx1*), Habib et al.^91^ identified an additional independent promoter for *slt-IB* in the upstream region of the subunit gene. Furthermore, they observed in addition to the bicistronic *slt-I* mRNA a monocistronic *slt-IB* mRNA that is most likely transcribed from this second promoter^91^. Contrarily, Sung et al.^66^ performed similar experiments using *slt-II* (syn. *stx2*) encoding strains and were not able to identify a monocistronic *slt-IIB* mRNA or an independent promoter for the B-subunit gene. However, they reported a second promoter for the *slt-II *operon 118 bases upstream of the *slt-IIA* gene^66^. Based on these observations, the question arises whether there exist additional promoters putatively varying in strength that influence the differences in transcription levels of the subunit genes in the six wildtype strains investigated in this study. However, Wagner et al.^55^ showed in their study by deletion of the antiterminator Q—pR’ region that pR’ is nonetheless the promoter which most strongly influences the Stx2 production. Besides additional promoters, the stability of the mRNA transcripts might affect the transcription levels of *stxA2a* and *stxB2a*. In *E. coli* the median mRNA half-life is ~ 3 min, when bacteria are grown at optimal conditions^92,93^. However, several studies reported the stabilization of overall mRNA or specific transcripts as a consequence on various stressors such as anaerobic conditions, carbon starvation and the stationary phase^93–96^.

At translational level, small regulatory RNAs (srRNA) or microRNAs might influence the protein levels of the single subunits. Nejman-Fulenczyka et al.^97^ identified in their study a 20-nt microRNA after induction of the Stx2-converting bacteriophage ϕ24B. This microRNA decreased the efficiency to lysogenize and led to a faster prophage induction after activation of the bacterial SOS response^97^. Sy et al.^76^ reported a srRNA in the *stx*-operon of both Stx1- and Stx2-converting bacteriophages which is processed from the transcript that arises in absence of the antiterminator Q and represses Stx1 production under lysogenic conditions. Since such antiterminated phage promoters are a generous source of srRNAs, the presence and effect of srRNAs should be also investigated in further studies using the six wildtype strains investigated in this study. Additional factors on translation level influencing the translation efficiency are the mRNA secondary structure and the accessibility of ribosome binding sites (RBS). Concerning those factors, Betley et al.^98^ reviewed for the cholera toxin, another AB_5_ protein toxin, that the RBS adjacent to *ctxB* is more efficiently than the RBS upstream of *ctxA* and that differences in the secondary structure of the *ctx*-mRNA between the A- and B- subunit regions could also result in unequal translation rates. For *slt-I*, similar results were observed by Habib et al.^91^. In their study, they identified a stem-loop structure surrounding the *slt-IB* RBS that influenced the level of B-subunit production, but no potential for secondary structure-formation in the nucleotides upstream of *slt-IA*^91^. However, by computer analysis, they also showed that the mRNA sequence surrounding the *slt-IIB* gene RBS has no potential for formation of a stem-loop structure^91^. Nevertheless, the mechanisms involved in the control of translation initiation on polycistronic transcripts are still unknown, but there is evidence that both, translation initiation and re-initiation is dependent on mRNA secondary structure^99^.

To be able to elucidate the mechanisms responsible for the strain-specific Stx expression and translation observed in this study and to clarify whether free A-subunits are involved in Stx-mediated pathogenicity of STEC and EHEC strains, further studies focusing on the genomic features involved in Stx transcription and translation as well as on factors determining translation efficiency are needed. These findings should help to get a more global understanding about the genetic and protein-based background of Stx production and to overcome the limitations of this study caused by the unique profiles of the wildtype strains used in this study.

## Materials and methods

### Bacterial strains

Bacterial strains used in this study are shown in Table 2. Strains were grown in Luria–Bertani (LB) broth (1.0% (w/v) tryptone, 0.5% (w/v) yeast extract, 1.0% (w/v) sodium chloride, adjusted to pH 7.0). Preparation of overnight cultures was performed by inoculating 10 mL LB broth (in 100 mL Erlenmeyer flask) with a single colony of the respective strain. Overnight cultures were incubated at 37 °C and 180 rounds per minute for ~ 16 h.

**Table 2:** *E. coli* strains used in this study.

Strain	Pathotype	Serotype	Relevant genotype	Reference
HUSEC003	EHEC	O157:H7	*stx2a*^+^, *subAB*^*−*^, *eae*^+^, *hlyA*^+^, *efa1*^+^, *iha*^+^, *sfpA*^-^, *lpf*O26^*−*^, *lpf*O113^*−*^, *lpf*O157-OI141^+^, *lpf*O157-OI154^+^, *saa*^*−*^, *terE*^+^	^67^
HUSEC004	EHEC	O157:H^*−*^	*stx2a*^+^, *subAB*^-^, *eae*^+^, *hlyA*^+^, *efa1*^+^, *iha*^*−*^, *sfpA*^+^, *lpf*O26^*−*^, *lpf*O113^*−*^, *lpf*O157-OI141^+^, *lpf*O157-OI154^+^, *saa*^*−*^, *terE*^*−*^	^67^
HUSEC008	EHEC	O103:H^*−*^	*stx2a*^+^, *subAB*^*−*^, *eae*^+^, *hlyA*^*−*^, *efa1*^+^, *iha*^*−*^, *sfpA*^*−*^, *lpf*O26^+^, *lpf*O113^*−*^, *lpf*O157-OI141^*−*^, *lpf*O157-OI154^*−*^, *saa*^*−*^, *terE*^*−*^	^67^
HUSEC018	EHEC	O26:H11	*stx2a*^+^, *subAB*^*−*^, *eae*^+^, *hlyA*^+^, *efa1*^+^, *iha*^+^, *sfpA*^*−*^, *lpf*O26^+^, *lpf*O113^+^, *lpf*O157-OI141^*−*^, *lpf*O157-OI154^*−*^, *saa*^*−*^, *terE*^+^	^67^
LB226692	EHEC	O104:H4	*stx2a*^+^, *subAB*^*−*^, *eae*^*−*^, *hlyA*^*−*^, *iha*^+^, *sfpA*^*−*^, *lpf*O26^+^, *lpf*O113^+^, *lpf*O157-OI141^*−*^, *lpf*O157-OI154^*−*^, *saa*^*−*^, *terE*^+^, *aatA*^+^, *aggA*^+^, *aggR*^+^, *set1*^+^, *pic*^+^	^85^
TS18/08	STEC	O113:H21	*stx2a*^+^, *subAB*^+^, *eae*^-^, *hlyA*^+^, *efa1*^*−*^, *iha*^+^, *sfpA*^*−*^, *lpf*O26^+^, *lpf*O113^+^, *lpf*O157-OI141^*−*^, *lpf*O157-OI154^*−*^, *saa*^+^, *terE*^+^	^7^
C600(933W)	K-12 derivate		*F-, λ-, thr-1, leuB6, lacY1, supE44, rfbD1, thi-1, mcrA1, cyn-1,* carrying the Stx2a-encoding prophage 933W	^100^

### RNA isolation

Twenty-four mL LB broth in a 250 mL Erlenmeyer flask was inoculated with an overnight culture of the respective strain to an initial optical density measured at 600 nm (OD_600nm_) of 0.05. Two main cultures were prepared per strain and incubated at 37 °C with 180 rounds per minute until an OD_600nm_ of ~ 0.3 was obtained. At an OD_600nm_ of 0.3, one culture was induced with 100 ng/mL norfloxacin using a 1.25 mg/mL norfloxacin stock solution (Sigma-Aldrich). The other culture was not induced and used as control. After further 2 h of incubation at 37 °C with 180 rounds per minute, the final OD_600nm_ was measured and the samples were adjusted to an OD_600nm_ of 1.0 (~ 1 × 10^9^ cells) with LB broth. A volume of 500 µL of the adjusted cell suspension was transferred to 1 mL of RNAprotect Bacterial Reagent (QIAGEN). The suspensions were mixed for 5 s and incubated at room temperature for 5 min. Centrifugation of the suspensions was performed at 5000 g for 10 min and the supernatants were discarded. The pellets were stored at − 70 °C until further processing. RNA isolation and purification were performed according to the manufacturer’s manual of the RNAprotect Bacterial Reagent (January 2020, QIAGEN), RNeasy Mini Kit (October 2019, QIAGEN) and RNase-free DNase Set (June 2018, QIAGEN), following the protocol for enzymatic lysis and proteinase K digest (protocol 4), subsequent purification of total RNA (protocol 7) and 2-times on column DNase digestion (appendix B). The concentration and purity of the RNA was determined using a NanoDrop 2000 spectrophotometer (Thermo Fisher Scientific) and RNA integrity was verified using denaturing agarose gel electrophoresis.

### DNase digest of isolated RNA and cDNA synthesis

Prior to complementary DNA (cDNA) synthesis, residual DNA was degraded by use of the DNA-free Kit (Thermo Fisher Scientific) following the manufacturer’s manual (Publication Number 1907M, Revision G). Reverse transcription was performed using iScript Select cDNA Synthesis Kit (Bio-Rad Laboratories) and a 1:1 blend of oligo(dT)_20_ and random primer. For this purpose, 500 ng of isolated RNA was used and incubated according to the manufacturer’s manual. To detect residual DNA in the further procedure, a reverse transcription-negative control was prepared for each of the samples. The concentration and purity of the cDNA was determined with a NanoDrop 2000 spectrophotometer (Thermo Fisher Scientific).

### Transcriptional analysis of Stx subunit gene expression

Expression of the Stx subunit genes *stxA2a* and *stxB2a* was investigated by transcriptional analysis using a CFX96 system (Bio-Rad Laboratories) following the procedure described previously by Hauser et al.^86^. Briefly, 10 µL SsoAdvanced universal SYBR green supermix (Bio-Rad Laboratories), 2.8 ng of cDNA, 0.75 µL of each primer (10 pmol/µL), and nuclease-free water (Thermo Fisher Scientific) to a final volume of 20 µL were used to prepare the samples for qPCR. Oligonucleotides and respective PCR program are shown in Table 3. For data analysis, the 2^−ΔΔCT^ method described by Pfaffl^101^ was used. For this purpose, gene expression of *stxA2a* and *stxB2a* was normalized to expression of the endogenous control gene *rrsB*. The ΔC_T_ values of samples of induced culture were normalized to those of the samples of the non-induced culture (ΔΔC_T_) using the following formula,

$$Relative normalized \;expression = \frac{{E_{{{\text{GOI}}}}^{{\Delta C_{T} {\text{GOI}}}} }}{{E_{{{\text{HKG}}}}^{{\Delta C_{T} {\text{HKG}}}} }}$$

where *E* is the converted primer efficiency, *C*_*T*_ is the threshold cycle, GOI is the gene of interest and HKG is the housekeeping gene.

**Table 3:** Oligonucleotides used in this study with respective PCR product size and PCR conditions.

oligonucleotide	nucleotide sequence (5′-3′ direction)	PCR product size	PCR conditions
rrsB-for	GCATAACGTCGCAAGACCAAA	91 bp	95 °C, 10 s; 60.5 °C, 30 s; 72 °C, 10 s; 40 cycles
rrsB-rev	GCCGTTACCCCACCTACTAGCT		
upper-stx	TCATATCTGGCGTTAATGGAGTTC	99 bp	95 °C, 10 s; 52.4 °C, 30 s; 72 °C, 10 s; 40 cycles
lower-stx	GCGTAAGGCTTCTGCTGTG		
stxB2-for	TGAAGAAGATGTTTATGGCGG	183 bp	95 °C, 10 s; 55.8 °C, 30 s; 72 °C, 10 s; 40 cycles
stxB2-rev	GAGCACTTTGCAGTAACGG		

The data were analyzed for each strain independently. For all target genes, standard curves were used to determine amplification efficiency (Table S3). For this purpose, a decimal dilution series of cDNA from the strain *E.* *coli* C600(933W) with concentrations between 0.0014 and 14 ng/µL was prepared and used as template in qPCR approach. No-template controls and reverse transcription-negative controls were used in each approach and showed C_T_ values of 34–40 (data not shown). Transcriptional analysis for each strain was performed in technical duplicates, and RNA isolation was performed in three biological replicates.

### Collection of culture supernatants

For the detection and quantification of free Stx subunits in the culture supernatants of the STEC and EHEC wildtype strains (Table 2), cultures (25 mL LB broth in 250 mL Erlenmeyer flask) were inoculated with overnight cultures of the respective strains to an initial OD_600nm_ of 0.05 and grown at 37 °C and 180 rounds per minute to an OD_600nm_ of ~ 0.3. At an OD_600nm_ of 0.3, cultures were induced with norfloxacin (100 ng/mL f.c.) (Sigma-Aldrich). After induction, strains were incubated for further 24 h (37 °C, 180 rounds per minute) and culture supernatants were collected and sterile filtered using a 0.22 µm PVDF syringe filter (Carl Roth). For subsequent native PAGE and Western blot analysis, culture supernatants were concentrated with Amicon Ultra Centrifugal Filter Units (MWCO: 3000 Da, Merck) to a final volume of 5 mL. For the determination of the number of subunits in the culture supernatant, unconcentrated samples were used. Culture supernatants of the STEC and EHEC wildtype strains were collected in three biological replicates.

### Native PAGE and Western blot analysis

To detect free Stx subunits in the culture supernatants, native polyacrylamide gel electrophoresis (nPAGE) with subsequent Western blot analysis was performed. For this purpose, culture supernatants and purified StxA2a and StxB2a were loaded on Bolt 4–12% Bis–Tris Plus gels (Thermo Fisher Scientific), and electrophoresis was conducted at 200 V for 50 min. After electrophoresis, nPAGE gels were blotted at 50 mA for 1 h on a polyvinylidene fluoride (PVDF) immunoblot membrane (Bio-Rad Laboratories). After blotting, membranes were washed with TBS-T (0.1% (v/v) Tween-20 in TBS) and blocked with blocking buffer (5% (w/v) milk powder (Carl Roth) in TBS-T) for 4 h at room temperature, gently rocking. For detection of StxA2a, Shiga toxin 2A Monoclonal Antibody (11E10, 1:5000, Thermo Fisher Scientific) and horseradish conjugated secondary antibody goat anti-mouse IgG (H + L), (1:5000, Thermo Fisher Scientific) was used. StxB2a was detected using anti-SCH-SKY antibody (1:5000, Davids Biotechnologie) and horseradish conjugated secondary antibody goat anti-rabbit IgG(H + L) (1:5000, Thermo Fisher Scientific). The anti-SCH-SKY antibody was synthesized for this study based on the following StxB2a-specific amino acid sequence: SKYNEDDTFTVKVDGKEY. Prior to use the specificity of this antibody was tested (see supplementary material). Incubation with the primary antibodies was performed at 4 °C, overnight and incubation with the secondary antibodies at RT for 4 h. Detection was performed using a 1:10 dilution of the SuperSignal West Atto Ultimate Sensitivity Substrate (Thermo Fisher Scientific) and a ChemiDoc XRS + device (Bio-Rad Laboratories) and the Image Lab Software for PC Version 6.1 (Bio-Rad Laboratories, https://www.bio-rad.com/de-de/product/image-lab-software?ID=KRE6P5E8Z#fragment-6).

### Stx-ELISA

For quantification of the number of StxA2a and StxB2a a quantitative ELISA was established. This ELISA was based on immobilized subunits being bound by the capture antibody and a horseradish conjugated detection antibody. The establishment of this ELISA is described in the supplementary material. For coating with the StxA2a and StxB2a standard, stock solutions with a final concentration of 1000 ng/mL were prepared using purified subunits. The purified subunits were obtained as described by Heinisch and Krause et al.^63^. Briefly, StxA2a was recombinantly expressed and purified using *E. coli* strain BL21(DE3) C41/pET45b(+)-s*txA2a* and anion exchange chromatography with a CaptoQ column for purification. For StxB2a, *E. coli* strain BL21(DE3) C41/pET22b(+)-*stxB2a* and immobilized metal affinity chromatography with a HisTrap HP column were used. The concentrations of the purified subunits were determined by Bradford assay as previously described by Bradford^102^. Briefly, the protein assay dye (Bio-Rad Laboratories) and a standard curve of bovine serum albumin (BSA; Carl Roth) was used. Detection was performed at 595 nm using a Tecan Infinite M200 device (Tecan).

StxA2a and StxB2a stock solutions were serial diluted (1:2) with 1 × PBS to obtain a protein standard in a range of 7.8 ng/mL and 1000 ng/mL. Coating of the 96-well plates (2HB, Thermo Fisher Scientific) was performed at 4 °C, overnight, gently rocking using the StxA2a and StxB2a standard, the culture supernatants of the STEC and EHEC wildtype strains in an appropriate dilution, and a PBS blank. To ensure that the Stx subunits are present in all samples in their monomeric form both, StxA2a and StxB2a stock solutions and the culture supernatants were denatured at 80 °C for 1 h prior before use. After the 96-well plates were washed three times with PBS-T (0.1% (v/v) Tween20 in PBS), 200 µL of blocking buffer (5% (w/v) milk powder (Carl Roth) in PBS-T) was added and the plates were incubated at room temperature for 1 h. Subsequently, plates were again washed three times with PBS-T and 100 µL of the respective antibody was added. For detection of StxA2a, Shiga toxin 2A Monoclonal Antibody (11E10, 1:1000) and horseradish conjugated secondary antibody goat anti-mouse IgG (H + L), (1:5000) was used. StxB2a was detected using anti-SCH-SKY antibody (1:1000) and horseradish conjugated secondary antibody goat anti-rabbit IgG(H + L) (1:5000). Incubation with the primary and secondary antibody was performed at room temperature for 2 h. In between and after incubation with the secondary antibody, plates were washed three times with PBS-T. For detection, a 1:10 dilution of the SuperSignal™ ELISA Femto Substrate (Thermo Fisher Scientific) and a Tecan Infinite M200 device (Tecan) was used. Chemiluminescent signal was measured at 428 nm, after shaking the plate for 60 s (orbital, amplitude 2). Quantification of the number of subunits was performed for each strain in three biological replicates with two technical replicates.

### Statistical analysis

For transcriptional analysis and Stx-ELISA, means calculated from the three biological replicates were used for statistical analysis. Data were analyzed for normal distribution using the Shapiro–Wilk test. To compare the datasets for the different strains, one-way analysis of variance (ANOVA) was performed using Tukey test and Bonferroni test (*p* = 0.01). The homogeneity of variance was tested using Levene’s test (*p* = 0.01).

## Electronic supplementary material

Below is the link to the electronic supplementary material.

Supplementary Material 1

## Data Availability

All data generated or analyzed during this study are included in this published article and its supplementary information files. Bacterial strains are available from the authors. Contact: Professor Dr. Herbert Schmidt, Institute of Food Science and Biotechnology, Department of Food Microbiology, University of Hohenheim, Garbenstraße 28, 70599 Stuttgart, Germany; herbert.schmidt@uni-hohenheim.de; phone: + 49 711 459 22305.
