# Supplementary material for: Excess A-subunits of Shiga toxin 2a are produced in enterohemorrhagic Escherichia coli
Source: Sci Rep. 2025 May 14;15:16712. doi: 10.1038/s41598-025-01342-2 (PMC12078605; doi:10.1038/s41598-025-01342-2)
Supplement: Supplementary file 1 — Supplementary Material 1 [file 41598_2025_1342_MOESM1_ESM.docx]

**Supplementary information**

**Excess A-subunits of Shiga toxin 2a are produced in enterohemorrhagic *Escherichia coli***

Katrin Neudek^1^, Theresa Kunz^1^, Holger Barth^2^, and Herbert Schmidt^1*^

^1^Department of Food Microbiology and Hygiene, Institute of Food Science and Biotechnology, University of Hohenheim. Garbenstrasse 28, 70599 Stuttgart, Germany

^2^Institute of Experimental and Clinical Pharmacology, Toxicology and Pharmacology of Natural Products, University of Ulm Medical Center, Albert-Einstein-Allee 11, 89081 Ulm, Germany

^*^To whom correspondence should be addressed:

Herbert Schmidt: [herbert.schmidt@uni-hohenheim.de](mailto:herbert.schmidt@uni-hohenheim.de)

**Raw values of the data shown in Figure 2 and Figure 4**

Table S1: mRNA fold-change and subunit ratio of *stxA2a* and *stxB2a* in *E. coli* strains TS18/08, LB226692, HUSEC003, HUSEC004, HUSEC008, and HUSEC018.

|  | mRNA fold-change [-]* | | expression ratio [-]* |
| --- | --- | --- | --- |
| strain | *stxA2a* | *stxB2a* | *stxA2a*:*stxB2a* |
| TS18/08 | 60.77 ± 22.16 | 25.32 ± 9.50 | 2.42 ± 0.20 |
| LB226692 | 6.87 ± 4.32 | 5.34 ± 2.67 | 1.22 ± 0.16 |
| HUSEC003 | 10.47 ± 1.88 | 8.20 ± 2.26 | 1.33 ± 0.22 |
| HUSEC004 | 263.25 ± 78.03 | 106.94 ± 41.75 | 2.60 ± 0.37 |
| HUSEC008 | 2.60 ± 1.39 | 1.69 ± 1.00 | 1.58 ± 0.28 |
| HUSEC018 | 461.69 ± 245.73 | 204.01 ± 111.67 | 2.26 ± 0.16 |

*Gene expression was investigated under inducing (100 ng/mL norfloxacin) and non-inducing growth conditions using qPCR. Quantification was performed using *rrsB* as an endogenous control. Expression ratio was calculated from the mRNA fold-change of *stxA2a* and *stxB2a*. Data are means ± standard deviation of the experiments performed in three biological replicates (n=3).

Table S2: Subunit ratio of StxA2a and StxB2a in *E. coli* strains TS18/08, LB226692, HUSEC003, HUSEC004, HUSEC008, and HUSEC018.

|  | subunit ratio [-]* |
| --- | --- |
| strain | StxA2a:StxB2a |
| TS18/08 | 4.19 ± 0.59 |
| LB226692 | 4.88 ± 0.86 |
| HUSEC003 | 1.12 ± 0.37 |
| HUSEC004 | 1.34 ± 0.30 |
| HUSEC008 | 0.85 ± 0.12 |
| HUSEC018 | 4.82 ± 1.47 |

*Subunit ration was calculated from the number of subunits of StxA2a and StxB2a, investigated under inducing growth conditions (100 ng/mL norfloxacin) using quantitative ELISA. Quantification was performed using a StxA2a and StxB2a standard within the range of 7.85 ng/mL and 1000 ng/mL. Data are means ± standard deviation of the experiments performed in three biological replicates (n=3).

**Raw data of the transcriptional analysis**

Table S3: Amplification efficiencies and converted amplification efficiencies of the target genes for qPCR.

| target gene | amplification efficiency [%]* | converted amplification efficiency [-] |
| --- | --- | --- |
| *rrsB* | 112.6 | 2.126 |
| *stxA2a* | 119.7 | 2.197 |
| *stxB2a* | 97.6 | 1.976 |

* Amplification efficiency was determined using a decimal dilution series of cDNA from strain *E. coli* C600(933W) with concentrations between 0.0014 to 14 ng/µL as template in the qPCR approach.

**
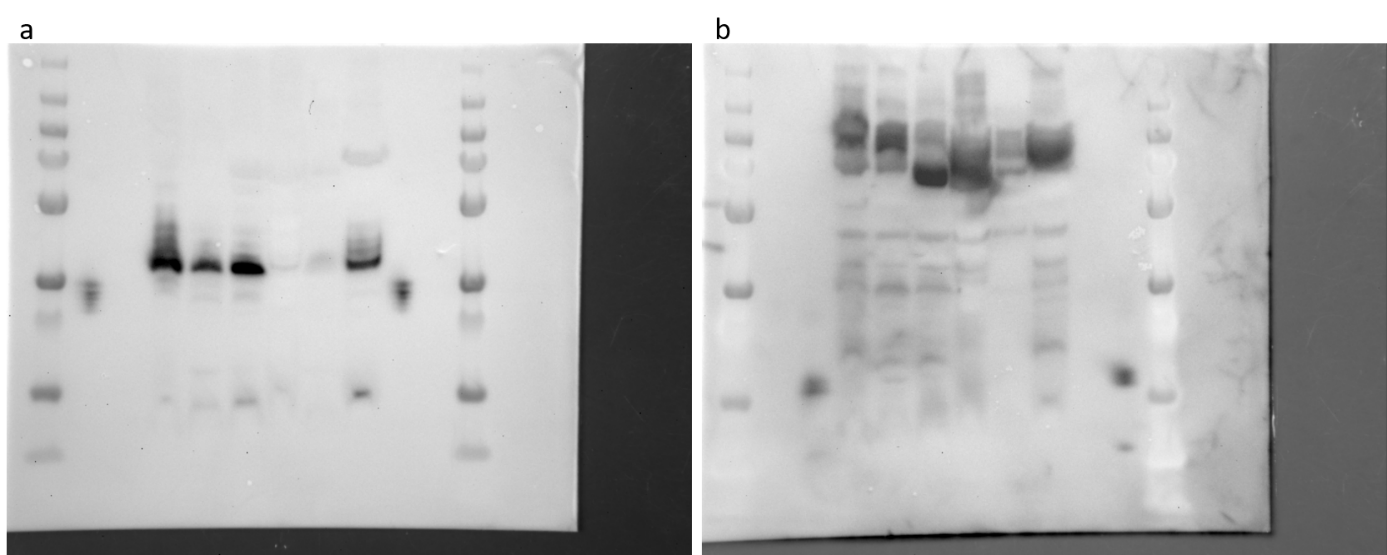
Uncropped results of Western blot analysis (Fig. 3)**

Figure S1: Uncropped images of Western blot analysis of culture supernatants of *E. coli* strains TS18/08, LB226692, HUSEC003, HUSEC004, HUSEC008, and HUSEC018. Samples were collected 24 h after induction with norfloxacin (100 ng/mL norfloxacin) and concentrated using centrifugal filter units (MWCO: 3,000 Da). PageRuler Plus Prestained Protein ladder (Thermo Fisher Scientific), purified StxA_2_ and StxB_2_ and concentrated culture supernatants were applied to Bolt 4-12 % Bis-Tris Plus gels. Western blot was detected with subunit-specific antibodies (a) Shiga toxin 2A monoclonal antibody (11E10) and (b) anti-SCH-SKY antibody in a 1:5000 dilution, respectively.

**Investigation of specificity of antibodies used in the study**

To ensure specific binding of the anti-SCH-SKY antibody to StxB2a, specificity was investigated using the culture supernatant of the Stx2a-producing strain *E. coli* strain C600(933W) and cell lysate of the laboratory strain *E. coli* strain C600. For this purpose, overnight cultures were prepared and used to inoculated main cultures (25 mL LB broth in 250 mL Erlenmeyer flask) to an initial OD_600nm_ of 0.05. The cultures were grown at 37 °C and 180 rounds per minute to an OD_600nm_ of ~ 0.3. At an OD_600nm_ of ~ 0.3, both cultures were induced with norfloxacin (100 ng/mL f.c.) (Sigma-Aldrich). After induction, strains were incubated for further 24 h (37 °C, 180 rounds per minute) and culture supernatant of *E. coli* strain C600(933W) was collected and sterile filtered using a 0.22 µm PVDF syringe filter (Carl Roth). For the cell lysate of *E. coli* strain C600, culture was harvested and cells were lysed using sonication (10 s on, 20 s off, 6 cycles, 70 % amplitude). For subsequent native PAGE and Western blot analysis, culture supernatant and cell lysate were concentrated with Amicon Ultra Centrifugal Filter Units (MWCO: 3,000 Da, Merck) to a final volume of 500 µL.


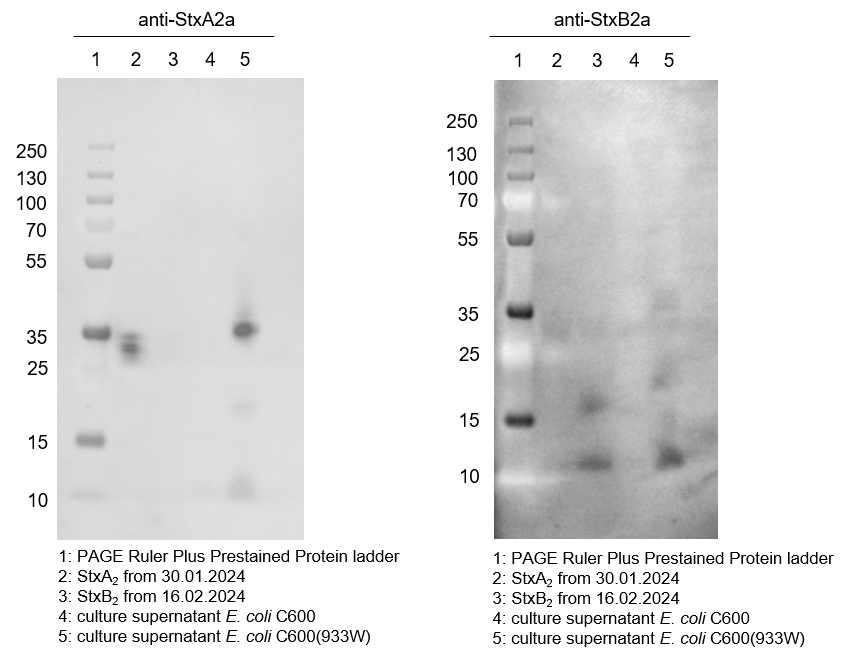
To investigate antibody specificity, native polyacrylamide gel electrophoresis (nPAGE) with subsequent Western blot analysis was performed. For this purpose, culture supernatant of *E. coli* strain C600(933W) and cell lysate of *E. coli* strain C600 and purified StxA2a and StxB2a were loaded on Bolt 4-12 % Bis-Tris Plus gels (Thermo Fisher Scientific), and electrophoresis was conducted at 200 V for 50 min. After electrophoresis, nPAGE gels were blotted at 50 mA for 1 h on a polyvinylidene fluoride (PVDF) immunoblot membrane (Bio-Rad Laboratories). After blotting, membranes were washed with TBS-T (0.1 % (v/v) Tween-20 in TBS) and blocked with blocking buffer (0.05 % (w/v) milk powder (Carl Roth) in TBS-T) overnight at 4 °C, gently rocking. For detection of StxA2a, Shiga toxin 2A Monoclonal Antibody (11E10, 1:5000, Thermo Fisher Scientific) and horseradish conjugated secondary antibody goat anti-mouse IgG (H+L), (1:5000, Thermo Fisher Scientific) was used. StxB2a was detected using anti-SCH-SKY antibody (1:5000, Davids Biotechnologie) and horseradish conjugated secondary antibody goat anti-rabbit IgG(H+L) (1:5000, Thermo Fisher Scientific). The anti-SCH-SKY antibody was synthesized for this study based on the following StxB2a-specific amino acid sequence: SKYNEDDTFTVKVDGKEY. Incubation with the primary antibodies was performed at 4 °C, for 2 h and incubation with the secondary antibodies at RT for 2 h. Detection was performed using a 1:10 dilution of the SuperSignal West Atto Ultimate Sensitivity Substrate (Thermo Fisher Scientific) and a ChemiDoc XRS+ device (Bio-Rad Laboratories) and the Image Lab Software for PC Version 6.1 (Bio-Rad Laboratories, <https://www.bio-rad.com/de-de/product/image-lab-software?ID=KRE6P5E8Z#fragment-6>).

Figure S2: Western blot analysis of culture supernatant of *E. coli* strains C600 and cell lysate of *E. coli* strain C600(933W). Samples were collected 24 h after induction with norfloxacin (100 ng/mL norfloxacin) and concentrated using centrifugal filter units (MWCO: 3,000 Da). PageRuler Plus Prestained Protein ladder (Thermo Fisher Scientific), purified StxA_2_ and StxB_2_ and concentrated culture supernatant and cell lysate were applied to Bolt 4-12 % Bis-Tris Plus gels. Western blot was detected with subunit-specific antibodies (Shiga toxin 2A monoclonal antibody (11E10) and anti-SCH-SKY antibody).

The Shiga toxin 2A monoclonal antibody (11E10) detected the single A-subunit (~ 35 kDa) in the culture supernatant of *E. coli* strain C600(933W) and no band in the cell lysate of *E. coli* strain C600 (Fig. S2, lane 4,5). Detection with the anti-SCH-SKY antibody revealed StxB monomers (~ 10 kDa), trimers (~ 20 kDa) and pentamers (~ 35 kDa) in the culture supernatant of *E. coli* strain C600(933W) and also no bands in the cell lysate of *E. coli* strain C600 (Fig. S2, lane 4,5). Thereby, the results of the Western blot analysis demonstrate, that neither the Shiga toxin 2A monoclonal antibody (11E10) nor the anti-SCH-SKY antibody showed any cross-reactivity with other proteins in *E. coli* cell lysates.

**Uncropped results of Western blot analysis (Fig. S2)**

**
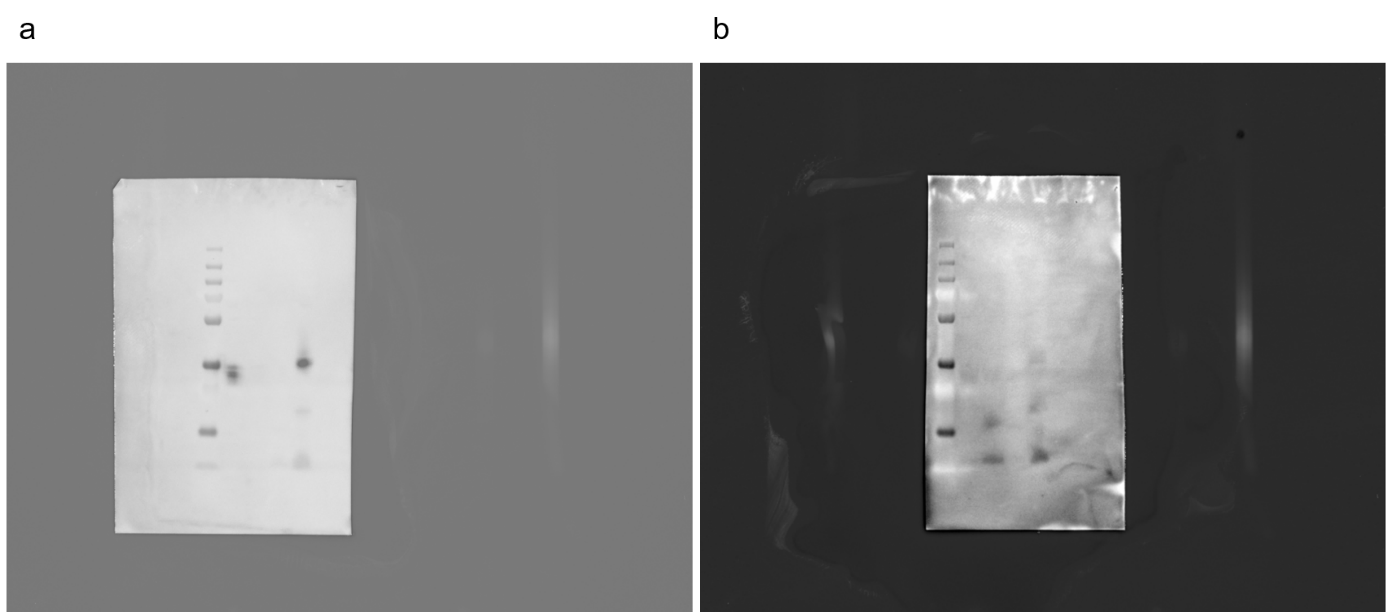
**Figure S3: Western blot analysis of culture supernatant of *E. coli* strains C600 and cell lysate of *E. coli* strain C600(933W). Samples were collected 24 h after induction with norfloxacin (100 ng/mL norfloxacin) and concentrated using centrifugal filter units (MWCO: 3,000 Da). PageRuler Plus Prestained Protein ladder (Thermo Fisher Scientific), purified StxA_2_ and StxB_2_ and concentrated culture supernatant and cell lysate were applied to Bolt 4-12 % Bis-Tris Plus gels. Western blot was detected with subunit-specific antibodies (Shiga toxin 2A monoclonal antibody (11E10) and anti-SCH-SKY antibody).

**Establishment of a quantitative ELISA specific for StxA2a and StxB2a**

A quantitative ELISA specific for StxA2a and StxB2a was established to determine subunit concentration and to calculate the number of subunits in the culture supernatants of STEC and EHEC wildtype strains. For detection of StxA2a, Shiga toxin 2A Monoclonal Antibody (11E10, Thermo Fisher Scientific) and horseradish conjugated secondary antibody goat anti-mouse IgG (H+L), (Thermo Fisher Scientific) was used. StxB2a was detected using anti-SCH-SKY antibody (Davids Biotechnologie) and horseradish conjugated secondary antibody goat anti-rabbit IgG(H+L) (Thermo Fisher Scientific). In the following, the primary antibodies are referred to as anti-StxA2a and anti-StxB2a. Since the primary antibody for detection of StxB2a is not a commercial antibody, an experimental set up for a correct application of anti-SCH-SKY was carried out (see below).

Determination of the optimal dilution of the capture and detection antibodies

In the first step, the optimal dilution of the primary and secondary antibody for detection of StxB2a was investigated. The aim was to determine a dilution in which the highest possible values for chemiluminescence could be measured. For this purpose, a dilution series of StxB2a within a concentration range from 4 µg/mL to 8 µg/mL was prepared using purified StxB2a and PBS and the quantitative ELISA was performed as described in the materials and methods section of this study. For detection, the primary antibody was tested in a 1:1000, 1:2000, and 1:5000 dilution. The secondary antibody was used in a 1:5000 dilution. All dilutions were prepared with PBS-T (0.1 % (v/v) Tween20 in PBS).

Figure S4: Chemiluminescence as a function of the concentration of StxB2a for the 1:1000 (white squares), 1:2000 (gray triangles), and 1:5000 (black dots) dilution of the primary antibody for detection of StxB2a as measured by ELISA.

The measured chemiluminescence showed highest values in a range from approximately 40,000 to 55,000 for the 1:1000 dilution of the primary anti-StxB2a antibody (Fig. S4). Dilutions 1:2000 and 1:5000 showed lower values between 40,000 and 48,000 (1:2000 dilution) and 28,000 und 34,000 (1:5000 dilution) (Fig. S4). Since the highest values and the largest differences between the values were obtained using the 1:1000 diluted primary antibody, this dilution was used in all further experiments. For the secondary antibody, the 1:5000 dilution was maintained. For detection of StxA2a, the same antibody dilutions were prepared.

Creation of a protein standard with linear correlation between concentration and chemiluminescence

In the second step, a protein standard based on a dilution series of purified StxB2a was created. This standard should have a linear correlation between protein concentration and measured chemiluminescence over a wide concentration range. For this purpose, a dilution series of purified StxB2a in a range of 50 ng/mL to 5000 ng/mL was prepared with PBS and used for coating of the ELISA plates.


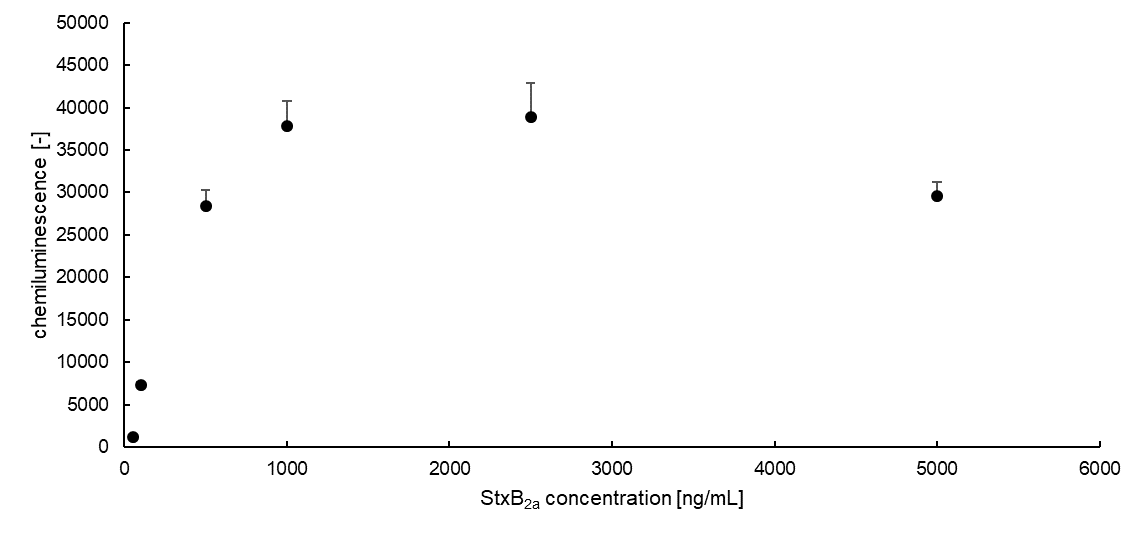
Figure S5: Standard curve of StxB2a over a concentration range from 5 ng/mL to 5000 ng/mL. Shown is the chemiluminescence as a function of the concentration of StxB2a. Data are means ± standard deviation of the experiments performed in two technical replicates (n=2).

The standard curve using a dilution series in the range of 50 ng/mL to 5000 ng/mL showed a linear correlation between concentration of StxB2a and chemiluminescence in a concentration range of 50 ng/mL to 1000 ng/mL (Fig. S2). At higher concentrations, no linear correlation could be observed (Fig. S5). Therefore, the concentration range of the standard was set to 31.3 ng/mL to 1000 ng/mL and serial dilution was prepared directly in the 96-well plate.

Figure S6: Standard curve of StxB2a over a concentration range from 31.3 ng/mL to 1000 ng/mL. Shown is the chemiluminescence as a function of the concentration of StxB2a with linear equation and the coefficient of determination (R^2^). Data are means ± standard deviation of the experiments performed in two technical replicates (n=2).

The standard curve using a dilution series in the range of 31.3 ng/mL to 1000 ng/mL showed a linear correlation between concentration of StxB2a and chemiluminescence over the entire concentration range with a R^2^ of 0.9944 (Fig. S6). In the next step, a standard in the same concentration range was prepared using purified StxA2a and tested in the ELISA.

Figure S7: Standard curve of StxA2a over a concentration range from 31.3 ng/mL to 1000 ng/mL. Shown is the chemiluminescence as a function of the concentration of StxA2a with linear equation and the coefficient of determination (R^2^). Data are means ± standard deviation of the experiments performed in two technical replicates (n=2).

Also, for StxA2a, the standard curve using a dilution series ranging from 31.3 ng/mL to 1000 ng/mL showed a linear correlation between the concentration of StxA2a and the chemiluminescence over the entire concentration range with an R^2^ of 0.9986 (Fig. S7).

In conclusion, a standard with a linear correlation between the measured chemiluminescence and the concentration of the respective subunit was created. This standard in the concentration range from 31.3 ng/mL to 1000 ng/mL was used to quantify the subunits in the culture supernatants of the STEC and EHEC wildtype strains.

Exclusion of cross-reactivity of the primary antibodies

In the last step, the cross-reactivity of the anti-StxA2a and anti-StxB2a antibody with the respective other subunit was investigated. For this purpose, detection of StxA2a was tested using the anti-StxB2a antibody and detection of StxB2a using the anti-StxA2a antibody. Both antibodies were diluted 1:1000 with PBS-T (0.1 % (v/v) Tween20 in PBS).


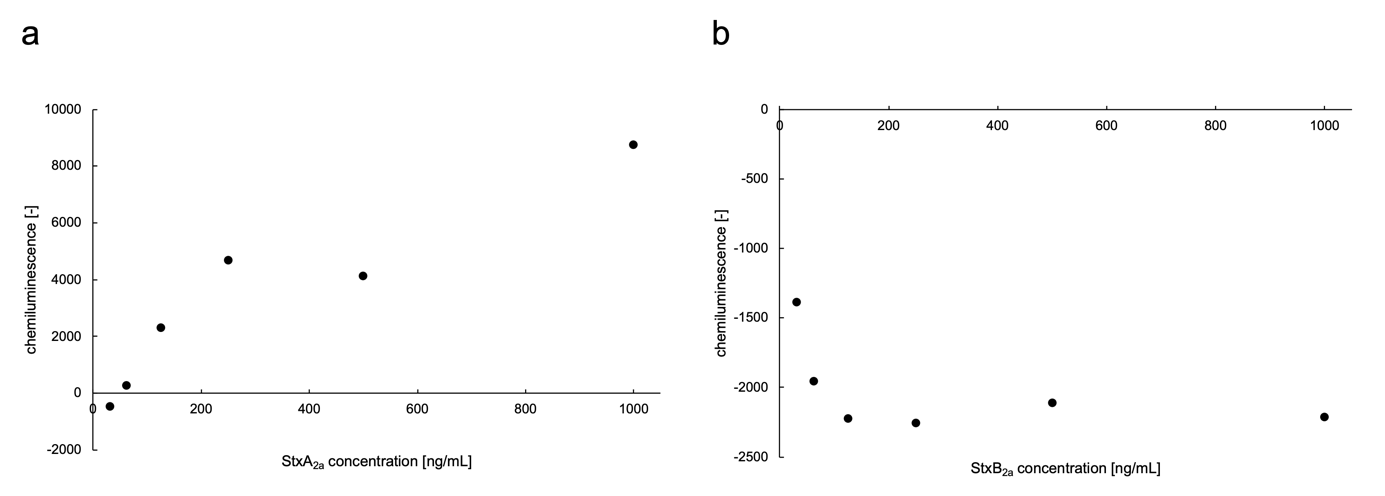


Figure S8: Chemiluminescence as a function of the concentration of StxA2a (a) and StxB2a (b) as measured by ELISA.

The chemiluminescence measured when detecting StxA2a with the anti-StxB2a antibody covered a range from approximately 480 at a concentration of 31.3ng/mL to approximately 9000 at a concentration of 1000 ng/mL (Fig. S8a). The measured values are therefore 8 - 22 times lower compared to detection of StxB2a with the same antibody (Fig. S8a). In addition, there is no continuous linear correlation between chemiluminescence and StxA2a concentration over the entire concentration range. Measurement of chemiluminescence when detecting StxB2a with the anti-StxA2a antibody revealed only negative values ranging from approximately -1400 at a concentration of 31.3 ng/mL to approximately -2250 at a concentration of 1000 ng/mL (Fig. S8b). In addition, also here no linear correlation was observed (Fig. S8b). Therefore, neither the anti-StxA2a nor the anti-StxB2a antibody showed cross-reactivity.
